# The MttB superfamily member MtyB from the human gut symbiont *Eubacterium limosum* is a cobalamin-dependent γ-butyrobetaine methyltransferase

**DOI:** 10.1016/j.jbc.2021.101327

**Published:** 2021-10-21

**Authors:** Jared B. Ellenbogen, Ruisheng Jiang, Duncan J. Kountz, Liwen Zhang, Joseph A. Krzycki

**Affiliations:** 1 Department of Microbiology, The Ohio State University, Columbus, Ohio, USA; 2 Campus Chemical Instrument Center Mass Spectrometry and Proteomics Facility, The Ohio State University, Columbus, Ohio, USA; 3 The Ohio State Biochemistry Program, The Ohio State University, Columbus, Ohio, USA

**Keywords:** bacterial metabolism, microbiology, energy metabolism, folate, microbiome, enzyme catalysis, cobalamin, one carbon metabolism, L-carnitine, γ-butyrobetaine, acetogenesis, ATCC, American Type Culture Collection, FDR, false discovery rate, QA, quaternary amine, THF, tetrahydrofolate, TMA, trimethylamine, TMAO, TMA *N*-oxide

## Abstract

The production of trimethylamine (TMA) from quaternary amines such as l-carnitine or γ-butyrobetaine (4-(trimethylammonio)butanoate) by gut microbial enzymes has been linked to heart disease. This has led to interest in enzymes of the gut microbiome that might ameliorate net TMA production, such as members of the MttB superfamily of proteins, which can demethylate TMA (*e.g.*, MttB) or l-carnitine (*e.g.*, MtcB). Here, we show that the human gut acetogen *Eubacterium limosum* demethylates γ-butyrobetaine and produces MtyB, a previously uncharacterized MttB superfamily member catalyzing the demethylation of γ-butyrobetaine. Proteomic analyses of *E. limosum* grown on either γ-butyrobetaine or dl-lactate were employed to identify candidate proteins underlying catabolic demethylation of the growth substrate. Three proteins were significantly elevated in abundance in γ-butyrobetaine-grown cells: MtyB, MtqC (a corrinoid-binding protein), and MtqA (a corrinoid:tetrahydrofolate methyltransferase). Together, these proteins act as a γ-butyrobetaine:tetrahydrofolate methyltransferase system, forming a key intermediate of acetogenesis. Recombinant MtyB acts as a γ-butyrobetaine:MtqC methyltransferase but cannot methylate free cobalamin cofactor. MtyB is very similar to MtcB, the carnitine methyltransferase, but neither was detectable in cells grown on carnitine nor was detectable in cells grown with γ-butyrobetaine. Both quaternary amines are substrates for either enzyme, but kinetic analysis revealed that, in comparison to MtcB, MtyB has a lower apparent *K*_*m*_ for γ-butyrobetaine and higher apparent *V*_max_, providing a rationale for MtyB abundance in γ-butyrobetaine-grown cells. As TMA is readily produced from γ-butyrobetaine, organisms with MtyB-like proteins may provide a means to lower levels of TMA and proatherogenic TMA-*N*-oxide *via* precursor competition.

The MttB superfamily comprises thousands of proteins found in bacteria and archaea. The first functionally described member is the namesake, MttB, the trimethylamine (TMA) methyltransferase, which initiates methanogenesis from that substrate by the methylation of a Co(I)-corrinoid protein (1). The gene encoding MttB is notable for possessing an amber codon encoding the 22nd amino acid, pyrrolysine (1, 2, 3, 4). Most genes encoding members of the MttB superfamily lack a pyrrolysine codon (*i.e.*, are nonpyrrolysine MttB homologs), leaving their function(s) in question (5). Recent work has shown some of these proteins are quaternary amine (QA)-dependent methyltransferases. Each demethylates a specific QA such as glycine betaine (5, 6, 7), proline betaine (8), or carnitine (9) in order to methylate a cognate corrinoid protein. The methylated corrinoid protein is then used to methylate catabolic cofactors such as tetrahydrofolate (THF) in bacteria or coenzyme M in methanogenic archaea. While this has provided some insights into the functions of this highly diverse superfamily, what QAs, or other compounds, are substrates remains unknown.

QAs are ubiquitous in nature and consequently found in many components of a typical human diet (10, 11, 12, 13). For example, γ-butyrobetaine is found in ruminant meat and milk (14). While often necessary, nutrients, γ-butyrobetaine, and other QAs have also been linked to the onset of cardiovascular disease as they are precursors for TMA production by members of the gut microbiota (15, 16, 17). Once absorbed into the bloodstream, TMA is oxidized in the liver (18) to TMA *N*-oxide (TMAO). Levels of serum TMAO correlate with the onset of atherosclerosis and increased occurrence of adverse cardiovascular events (16, 17, 19), vascular inflammation (20), graft *versus* host disease (21), colorectal (22, 23) and liver (24) cancers, and increased risk of mortality in patients with kidney disease (25, 26), coronary artery disease (27), as well as myocardial infarction (28).

The wide-ranging impact of TMA and TMAO on human health has given new impetus to understanding microbial metabolism of QAs in the largely anoxic environment of the mammalian gut. A direct route to TMA production from choline is afforded by CutC, the choline-TMA lyase (29, 30, 31). TMA can also be directly produced from l-carnitine as mediated by CntAB, the l-carnitine monooxygenase (32, 33). However, most of the TMA produced in the gut from l-carnitine is made with the intermediacy of γ-butyrobetaine (15) (Fig. 1). l-carnitine reduction to γ-butyrobetaine provides an additional electron acceptor for anoxic metabolism in enteric bacteria such as *Escherichia coli* or *Proteus* sp. (11, 34, 35). γ-Butyrobetaine production is considered to be controlled by the activity of CaiC, an ATP-dependent betainyl-CoA ligase (36). l-carnitinyl-CoA is then dehydrated and reduced by CaiD and CaiA, respectively (35). The CoA transferase CaiB can produce the end product γ-butyrobetaine while reinitiating the pathway by formation of l-carnitinyl-CoA without further energy expenditure. Because of this pathway, as well as dietary sources (14), γ-butyrobetaine can be found at low millimolar concentrations in human feces (16).

**Figure 1:**
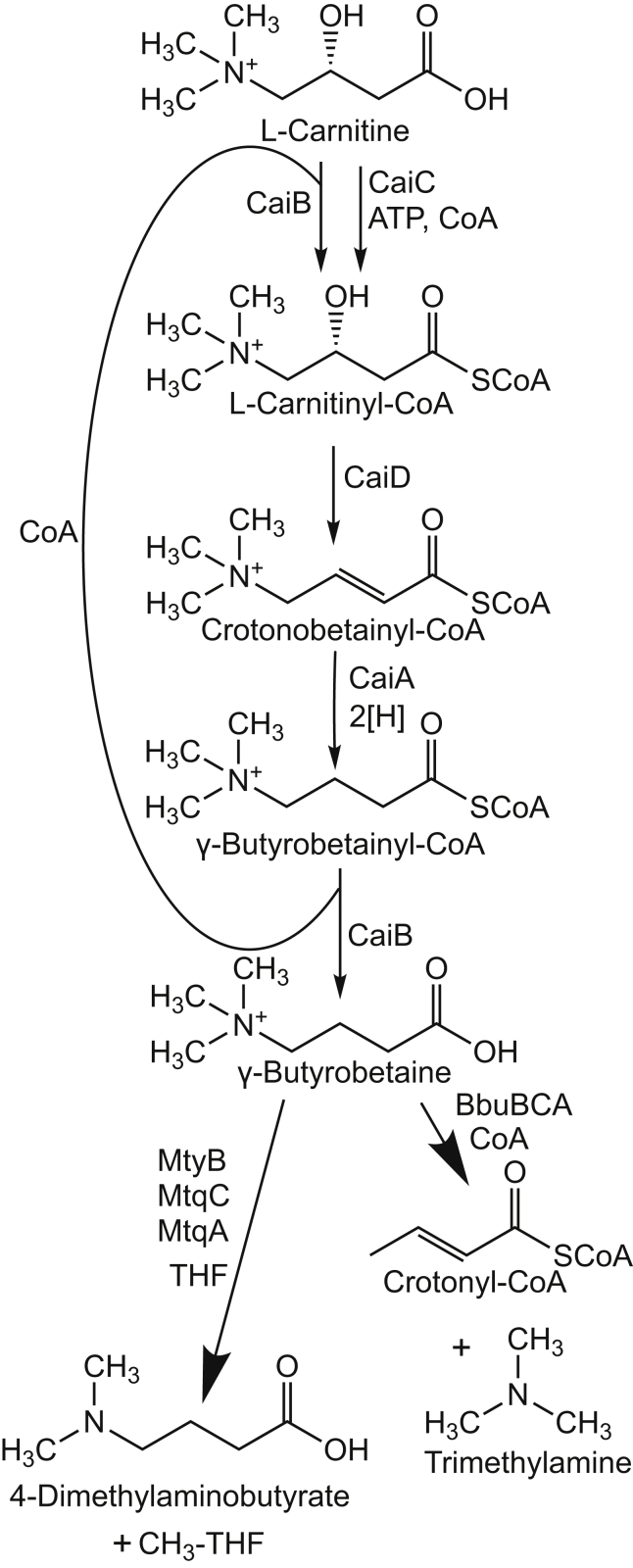
**γ-Butyrobetaine production and consumption by microbial enzymes in the human gut.** Under anaerobic conditions, enteric bacteria utilize dietary l-carnitine as an alternative electron acceptor using the indicated catabolic pathway. The excreted γ-butyrobetaine can be taken up and further metabolized by other members of the gut microbiome. Previously known catabolic enzymes that utilize γ-butyrobetaine produce proatherogenic TMA (*bottom right*). The enzyme system described here (*bottom left*) uses γ-butyrobetaine as a substrate but does not produce TMA. Further details can be found in the text. TMA, trimethylamine.

Both the oxygenases, YeaWX (15) and CntAB (32, 37), have been shown to cleave γ-butyrobetaine to TMA. However, it is not clear if these enzymes function to a significant extent in the largely anoxic intestine (38). Only recently was BbuA described, a flavoprotein that functions anoxically to cleave γ-butyrobetainyl-CoA to crotonyl-CoA and TMA (38). Highly similar homologs of *bbuA* are found in intestinal isolates as well as gut microbiota metagenomic datasets (38). The rise in TMAO levels after eating red meat is attributable to the l-carnitine/γ-butyrobetaine pathway (39). Increased serum levels of γ-butyrobetaine and l-carnitine in patients with carotid atherosclerosis have been associated with increased risk of cardiovascular mortality (40, 41, 42).

Net TMAO production relies on the amount of TMA produced by the microbiome. Net TMA production relies not only just on microbes producing TMA but also on those that consume the QAs that are the TMA precursors, as well as TMA itself. In this regard, the MttB superfamily occupies a unique position. Demethylation of TMA by MttB, the pyrrolysyl protein that is the founding member of the superfamily, has been suggested as a means to control TMA levels (43). The genomes of some intestinal isolates have also been found to encode MttB superfamily members that lack pyrrolysine, and several of these enzymes have been shown to demethylate QAs. For example, the acetogenic human gut symbiont *Eubacterium limosum* American Type Culture Collection (ATCC) 8486 produces MtpB (8) or MtcB (9), which demethylate proline betaine or l-carnitine, respectively, during methylotrophic growth on these substrates. Demethylation of a proatherogenic QA such as l-carnitine would render the product incapable of being used as a substrate for TMA production by any known enzyme. Members of the intestinal microbiota equipped with MttB superfamily members that demethylate proatherogenic QAs might serve as a natural governor to limit TMA production.

The genome of *E. limosum* ATCC 8486 (44, 45) encodes 40 additional nonpyrrolysine MttB homologs, suggesting significant uncharacterized potential for QA demethylation, possibly of major TMA precursors. While MtcB has such potential for l-carnitine, it must be recognized that most ingested l-carnitine is rapidly turned into γ-butyrobetaine by the gut microbiota (15). MtcB was previously found to have activity with γ-butyrobetaine as a methyl donor, but the activity was weak relative to its primary substrate, l-carnitine (9). However, the low activity of MtcB with γ-butyrobetaine provided an indication that an MttB superfamily member might carry out a robust demethylation of γ-butyrobetaine.

Here, we demonstrate for the first time that the human gut symbiont *E. limosum* ATCC 8486 is capable of growth by demethylating γ-butyrobetaine and describe a methyltransferase system that uses γ-butyrobetaine to methylate THF, forming a key catabolic intermediate of acetogenesis. Using a proteomics approach, we identified a previously uncharacterized nonpyrrolysine MttB homolog we designated MtyB. MtyB is highly abundant in *γ*-butyrobetaine-grown cells relative to cells grown on lactate, proline betaine, or l-carnitine, and acts as an *γ*-butyrobetaine:corrinoid protein methyltransferase. MtyB, along with an abundant corrinoid protein and THF-dependent methyltransferase, act as a γ-butyrobetaine:THF methyltransferase system. Michaelis–Menten kinetics comparing MtyB and the l-carnitine methyltransferase MtcB provide a physiological rationale for the observation that MtcB and MtyB are abundant only in cells grown l-carnitine or γ-butyrobetaine, respectively. Our results expand the substrate range of the MttB superfamily and show that a member of the gut microbiota can potentially intercept the l-carnitine-dependent pathway of TMAO production by consuming either l-carnitine or γ-butyrobetaine.

## Results

### *E. limosum* grows while concomitantly demethylating γ-butyrobetaine

**Figure 2:**
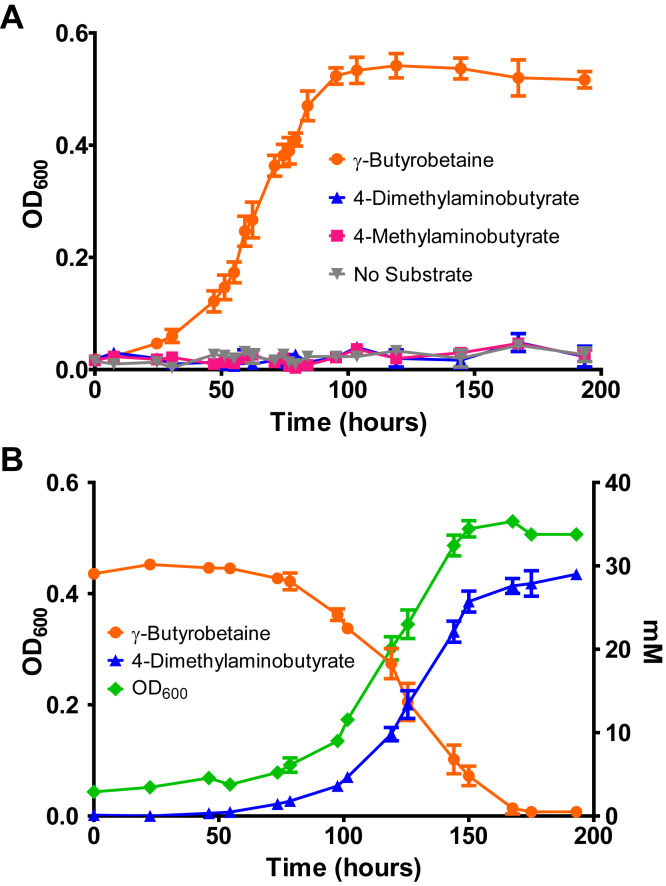
**Growth of *Eubacterium limosum* with γ-butyrobetaine occurs with simultaneous demethylation of the substrate to 4-dimethylaminobutyrate.***A*, *E. limosum* grows with γ-butyrobetaine in a defined medium but not in the absence of substrate or with 4-dimethylaminobutyrate or 4-methylaminobutyrate in place of γ-butyrobetaine. Three independent cultures were grown under a 100% nitrogen atmosphere with 30 mM of the indicated substrate. Error bars represent standard deviations of the mean absorbance readings at 600 nm. *B*, depletion of γ-butyrobetaine is coupled to the formation of 4-dimethylaminobutyrate during growth of *E. limosum*. Both compounds were quantified by ^1^H-NMR (n = 3) of the clarified supernatant from *E. limosum* cultures taken at indicated time points during growth with γ-butyrobetaine. Further demethylation products of γ-butyrobetaine were not detected. Each point on the graph represents the mean value from three independently grown γ-butyrobetaine-supplemented cultures. Error bars represent standard deviation.

*E. limosum* has been previously demonstrated to demethylate several QAs (8, 9, 46) but not the proatherogenic QA, γ-butyrobetaine. We found that when *E. limosum* ATCC 8486 is cultured with γ-butyrobetaine (Fig. 2*A*) in a defined medium growth occurred with a doubling time of 13 ± 1.5 h (n = 3). No growth was observed in the absence of substrate, or with either 4-dimethylaminobutyrate or 4-methylaminobutyrate (Fig. 2*A*), the sequential demethylation products of γ-butyrobetaine.

In order to confirm that γ-butyrobetaine was demethylated during growth on the substrate, samples were withdrawn daily from cultures in triplicate and subsequently analyzed by quantitative ^1^H-NMR (Figs. 2*B* and S1). The singlet peak at 3.12 ppm corresponding to ((CH_3_)_3_-N^+^-) was integrated to estimate γ-butyrobetaine concentrations, whereas the singlet peak at 2.88 ppm corresponding to the methyl protons of ((CH_3_)_2_-NH^+^-) was integrated to estimate the concentration of 4-dimethylaminobutyrate (Fig. S1). Although the doubling time (22 ± 2.4 h; n = 3) was slower, likely because of the sampling of the anaerobic culture, growth clearly coincided with the disappearance of γ-butyrobetaine, and the accumulation of 4-dimethylaminobutyrate in a 1:1 M ratio (Figs. 2*B* and S1). Further demethylation products of γ-butyrobetaine were not detectable. These results illustrate that *E. limosum* grows dependent on the presence of γ-butyrobetaine while catalyzing a single demethylation of this QA.

### Proteomic analysis of γ-butyrobetaine-grown *E. limosum*

We employed a quantitative proteomics approach to identify candidate proteins that might mediate the demethylation of γ-butyrobetaine and generate methyl-THF for acetogenesis. Catabolic enzymes are known to be abundant in anaerobic microbes, as the limited energy yields of their catabolic pathways often require high substrate turnover to maximize growth rate. We previously employed a similar proteomics approach to the identification of multicomponent methyltransferase systems from *E. limosum* capable of demethylation of other QAs such as proline betaine (8) and l-carnitine (9).

Lysates of cells harvested from four different cultures of *E. limosum* grown with γ-butyrobetaine to mid-log phase were each subjected to trypsin digestion and analyzed *via* LC–MS/MS. Detected peptides were mapped against the closed genome of *E. limosum* ATCC 8486 (45). Calculated exponentially modified protein abundance index scores (47) were then used to estimate the percent molar abundance of detected proteins. Approximately 1600 proteins were identified from cells grown on γ-butyrobetaine (Tables S1 and S2), similar to the number previously identified using the same procedures with lactate-grown cells (8, 9). As expected, enzymes of the central Wood–Ljungdahl pathway of acetogenesis were detectable in cells grown with γ-butyrobetaine at similar abundance to the levels observed in cells grown on lactate (Table S3). In addition, cells grown on γ-butyrobetaine either lacked or had markedly diminished levels of subunits of lactate dehydrogenase previously known to be abundant in lactate-grown cells (48); the subunits of which were abundant in the lactate-grown cell proteome (8, 9) (Table S4). Conversely, we identified several proteins that were more abundant in γ-butyrobetaine-grown cells relative to those grown on lactate whose characteristics indicated possible involvement in the early steps of methylotrophic acetogenesis with γ-butyrobetaine (Table 1). These included a nonpyrrolysine MttB superfamily member (WP_013382626.1), which we designated MtyB. MtyB constituted 0.9 ± 0.4 mol% from γ-butyrobetaine-grown cells but was not detectable in the proteome of cells grown with lactate. MtyB was also not detectable in the previously studied proteomes of cells grown with proline betaine or carnitine (8, 9). However, reminiscent of the genome context of MtcB (9), MtyB is encoded in the genome adjacent to a member of the major facilitator superfamily of transporters and divergently transcribed from an annotated FIS homolog homologous to RocR (Fig. S2), a regulator of cationic amino acid metabolism, a homolog of which is found divergently transcribed from MtcB.

**Table 1:** γ-butyrobetaine:THF methyltransferase system components found in *E. limosum* proteome

Name	Accession number	Mol % of total soluble protein observed in γ-butyrobetaine-grown cells	Mol % of total soluble protein observed in lactate-grown cells	Fold change^a^	*p*
MtyB	WP_013382626.1	0.88 ± 0.41	Not detected	≥8800^b^	0.0053
MtqC	WP_038352545.1	0.46 ± 0.26	0.0083 ± 0.0039	55	0.0150
MtqA	WP_038351870.1	1.9 ± 1.2	0.10 ± 0.032	18	0.0230
RamQ	WP_038351871.1	0.026 ± 0.0037	0.0094 ± 0.0034	2.8	0.0005

a Ratio of mol % protein in γ-butyrobetaine-grown *versus* DL-lactate-grown cells.

b A lower limit of detection of 0.0001% of total soluble protein was used to estimate this value.

Thus far, characterized members of the MttB superfamily have been shown to methylate either a supplied free corrinoid cofactor or a corrinoid protein (1, 5, 6, 7, 8, 9). Therefore, we hypothesized that MtyB might also be a corrinoid-dependent methyltransferase but with specificity for γ-butyrobetaine. Supporting this hypothesis, a corrinoid protein, MtqC, and a corrinoid:THF methyltransferase, MtqA, were also found to be abundant in γ-butyrobetaine-grown cells but significantly less abundant in those grown on lactate (Table 1). MtqC and MtqA were previously found to catalyze THF methylation when supplemented with either MtpB and proline betaine (8, 9) or MtcB and l-carnitine (8, 9). In addition, the ATP-dependent corrinoid protein reductive activase, RamQ, was also upregulated (Table 1). In summary, our proteomics approach led us to hypothesize that *E. limosum* ATCC 8486 possesses a γ-butyrobetaine-dependent methyltransferase system comprised of MtyB, MtqC, MtqA, and RamQ.

### Production of recombinant MtyB requires coproduction of recombinant MtqC

In order to begin testing if we had identified components of an *E. limosum* γ-butyrobetaine:THF methyltransferase system, the *mtyB* gene was cloned into *E. coli* for recombinant expression. However, multiple approaches failed to produce active MtyB and resulted in the production of insoluble protein, even when employing an *mtyB* gene that was codon optimized for expression in *E. coli* (Fig. S3). Finally, coexpression of the codon-optimized *mtyB* gene with the *mtqC* gene permitted soluble protein production of both proteins in *E. coli* Tuner(DE3). Furthermore, the hexahistidine-tagged MtyB and a protein corresponding to the size of apo-MtqC were found to coelute from a nickel affinity column (Fig. S4), despite only MtyB bearing a histidine tag for the column. As previously observed, apo-MtqC lacking cobalamin is produced in *E. coli* (8), but soluble expression of MtyB dependent on the coexpression of MtqC, coupled with the apparent coelution of MtyB and apo-MtqC from the nickel-affinity column, indicated possible interaction of the two proteins, even in the absence of the cofactor. MtyB and apo-MtqC were subsequently resolved with an anion exchanger to yield purified soluble MtyB (Fig. S5).

### MtyB is a γ-butyrobetaine:corrinoid protein methyltransferase

**Figure 3:**
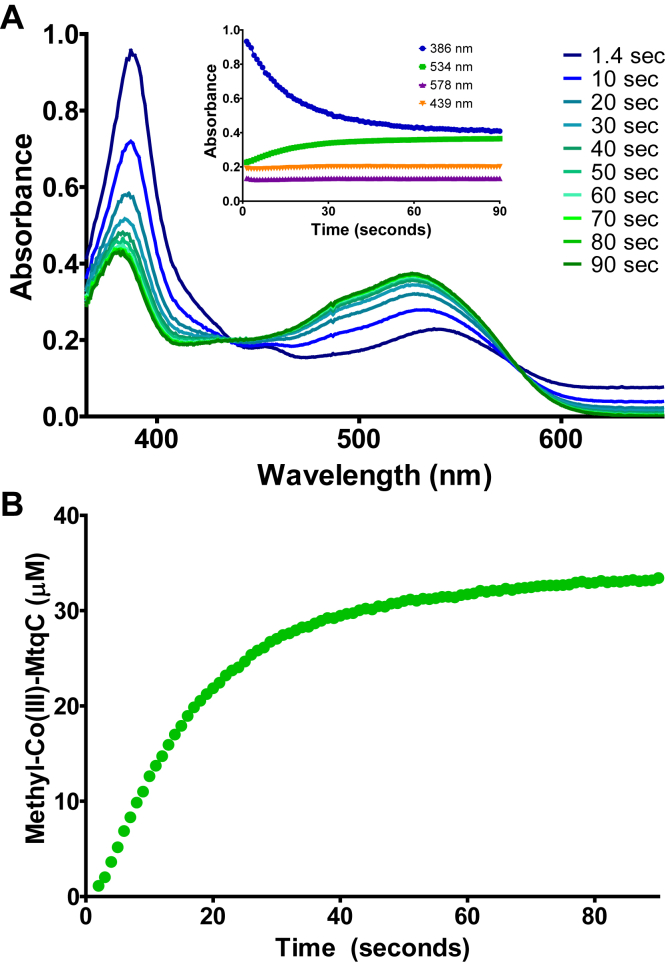
**MtyB acts as a γ-butyrobetaine:MtqC methyltransferase.***A*, representative UV–visible spectra collected at the indicated times during MtyB-catalyzed methyl group transfer from γ-butyrobetaine to Co(I)-MtqC in an anoxic cuvette. Co(II)-MtqC was reduced to the Co(I) state prior to initiation of the assay by addition of RamQ, ATP, and Ti(III)citrate. The decrease in the major peak at 386 nm corresponds to the disappearance of Co(I)-MtqC, whereas the increase in methyl-Co(III)-MtqC gives rise to the increased absorbance at 534 nm. Isosbestic points of the Co(I)-MtqC and methyl-Co(III)-MtqC spectra are found at 439 and 578 nm. The *inset* shows the individual absorbance traces at 386, 439, 534, and 578 nm during the course of the reaction. *B*, time-dependent formation of methyl-Co(III)-MtqC with γ-butyrobetaine as catalyzed by MtyB. The methylated form of the corrinoid protein was quantified using the aforementioned UV–visible spectra data and the previously determined extinction coefficients (8).

MtcB, the carnitine methyltransferase, has a robust l-carnitine:cob(I)alamin methyltransferase activity (9). Therefore, we first tested MtyB for activity as a γ-butyrobetaine:cob(I)alamin methyltransferase. However, in the presence of MtyB and γ-butyrobetaine, we could not detect methylation of cob(I)alamin. MtyB was next tested for γ-butyrobetaine:MtqC methyltransferase activity. MtqC was produced as described previously by Picking *et al.* (8). Purified MtqC holoprotein is initially in the inactive Co(II) oxidation state and was therefore reduced to the Co(I) state with the ATP-dependent reductive activase RamQ prior to assay. Upon initiation of the reaction by either MtyB or γ-butyrobetaine, Co(I)-MtqC was methylated as indicated by the decrease in absorbance at 386 nm and the increase in absorbance at 534 nm because of the conversion of Co(I)-MtqC to methyl-Co(III)-MtqC (Fig. 3).

**Figure 4:**
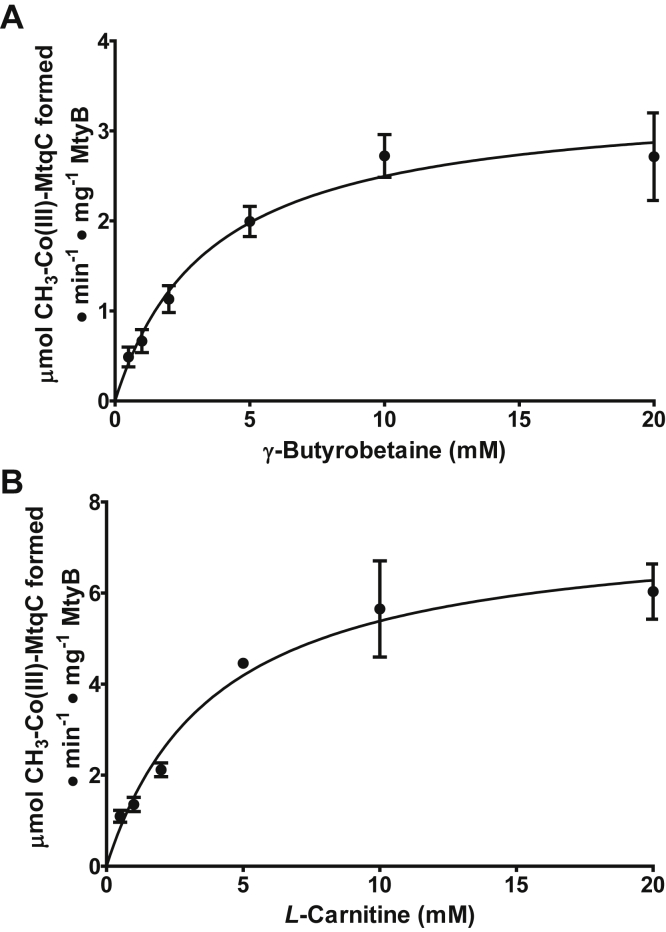
**Kinetic characterization of MtyB-catalyzed methyl-Co(III)-MtqC formation using either γ-butyrobetaine or****l****-carnitine as methyl donors.** Rates of Co(I)-MtqC methylation by MtyB were determined at increasing concentrations of (*A*) γ-butyrobetaine or (*B*) l-carnitine at a constant MtqC concentration. Points represent the mean initial rate of three reactions at each concentration with standard deviation shown by the error bars. Each line was plotted to the Michaelis–Menten equation using nonlinear regression.

The sharp isosbestic points at 439 and 578 nm indicated that methylation occurred without the significant accumulation of any other forms of MtqC, such as Co(II)-MtqC. The rate of the reaction was estimated using the extinction coefficients calculated by Picking *et al.* (8). MtyB followed Michaelis–Menten kinetics when the concentration of the QA was varied at a constant concentration of Co(I)-MtqC (Fig. 4*A*). Using nonlinear regression, MtyB was found to have an apparent *K*_*m*_ for γ-butyrobetaine of 3.5 ± 0.7 mM, similar to what has been noted for other QA-dependent MttB superfamily members (5, 8, 9). The apparent *V*_max_ for the reaction is 3.4 ± 0.2 μmol·min^−1^ mg^−1^ MtyB, equivalent to a *k*_cat_ of 180 ± 10 min^−1^.

Since γ-butyrobetaine is similar to l-carnitine, except for the lack of a β-hydroxyl group, MtyB was also tested for activity with l-carnitine. We found that MtyB catalyzes a robust l-carnitine:MtqC methyl transfer and displayed Michaelis–Menten kinetics (Fig. 4*B*) comparable to what was observed for the enzyme with γ-butyrobetaine. Using nonlinear regression, MtyB was found to have an apparent *K*_*m*_ for l-carnitine of 4.0 ± 0.7 mM. The *V*_max_ for the reaction is 7.5 ± 0.49 μmol·min^−1^ mg^−1^ MtyB, equivalent to a *k*_cat_ of 407 ± 26.5 min^−1^. MtyB catalyzed l-carnitine:MtqC methyl transfer slightly faster than γ-butyrobetaine:MtqC methyl transfer, albeit with a highly similar apparent *K*_*m*_. Calculation of the specificity constant of MtyB with γ-butyrobetaine (8.5 × 10^2^ M^−1^ s^−1^) and l-carnitine (1.7 × 10^3^ M^−1^ s^−1^) suggests that the two substrates are kinetically similar as methyl donors by MtyB.

MtyB is also capable of catalyzing the demethylation of other methylated amines; however, at much lower rates than observed with γ-butyrobetaine or l-carnitine as a substrate. MtyB methylated Co(I)-MtqC with 25 mM of the following QAs at the indicated rates, each measured in triplicate:glycine betaine, 14 ± 1.3 nmol·min^−1^ mg^−1^ MtyB; proline betaine, 12 ± 2.1 nmol·min^−1^ mg^−1^ MtyB; and choline, 19 ± 3 nmol·min^−1^ mg^−1^ MtyB. Activity with 4-dimethylaminobutyrate, the demethylation product of γ-butyrobetaine, was below the limit of detection. However, MtyB could methylate Co(I)-MtqC with 4-methylaminobutyrate at a rate of 25 ± 6 nmol·min^−1^ mg^−1^ MtyB. Notably, even though <1% of the activity with γ-butyrobetaine, this is the first time any nonpyrrolysine MttB superfamily member has shown activity with a secondary amine.

### MtcB, the l-carnitine methyltransferase from *E. limosum*, does not share the equal affinity of MtyB for both γ-butyrobetaine and l-carnitine

MtyB is abundant in cells grown with γ-butyrobetaine but was not previously identifiable in the proteome of *E. limosum* grown with l-carnitine (9). Conversely, MtcB, the l-carnitine:Co(I)-MtqC methyltransferase is not detectable in the γ-butyrobetaine proteome. However, a BLAST alignment of MtyB and MtcB showed that these two proteins share 53% identity and 70% similarity over 99% of both proteins (Fig. S6). Furthermore, a phylogenic analysis of the 42 MttB superfamily members in the *E. limosum* genome revealed that MtcB and MtyB are the two most similar MttB superfamily members in the *E. limosum* genome (Fig. S7). MtyB has similar kinetics with both γ-butyrobetaine and l-carnitine. Kountz *et al.* (9) previously reported the Michaelis–Menten kinetic parameters of MtcB with l-carnitine but not with γ-butyrobetaine. Kinetic analysis done under identical assay conditions as used previously estimated that MtcB has an apparent *K*_*m*_ for γ-butyrobetaine of 15 ± 6 mM and an apparent *V*_max_ of 150 ± 19 nmol·min^−1^ mg^−1^ MtcB (*k*_cat_ of 8.3 ± 1.1 min^−1^). The specificity constant of MtcB with l-carnitine using the published parameters is 9.3 × 10^4^ M^−1^ s^−1^ is four orders of magnitude higher than with γ-butyrobetaine (9.2 M^−1^ s^−1^), indicating that, unlike MtyB, MtcB differentiates between these two substrates with a strong preference for l-carnitine.

### *In vitro* reconstitution of the γ-butyrobetaine:THF methyltransferase system

**Figure 5:**
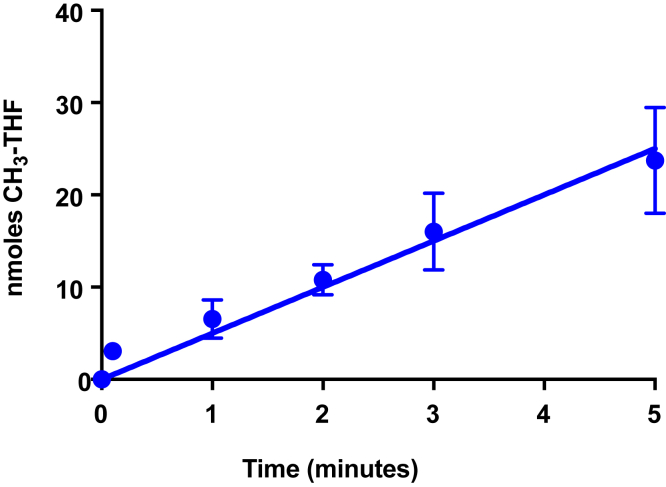
**Reconstitution of the γ-butyrobetaine:THF methyltransferase system *in vitro*.** MtyB, MtqC, and MtqA were incubated with γ-butyrobetaine in vials made anoxic with 100% nitrogen. Prior to initiation of the reaction, RamQ, ATP, and Ti(III)citrate were added, and reaction vials were preincubated at 37 °C for 5 min to reduce MtqC to the Co(I) state, and then the reaction was initiated by addition of THF. Methyl-THF formation was measured *via* HPLC analysis of time points. Each data point is the mean of methyl-THF formed in duplicate reactions with error bars representing range. No methyl-THF was detected in duplicate negative controls incubated for the same time, which were otherwise complete reactions except for the absence of MtyB, MtqC, MtqA, or γ-butyrobetaine. THF, tetrahydrofolate.

To our knowledge, an enzyme system has not previously been demonstrated to catalyze the methylation of THF with γ-butyrobetaine. Therefore, we conducted anoxic reactions in which MtyB, MtqC, and MtqA were incubated in the presence of γ-butyrobetaine. In order to reduce MtqC to the active Co(I) state, RamQ, MgATP, and Ti(III)citrate were incubated with the corrinoid protein and two methyltransferases prior to initiation of the reaction by addition of THF (Fig. 5). At the indicated time points, samples were removed and quenched with saturated trichloroacetic acid prior to analysis using reverse-phase HPLC. We observed an initial rate of methyl-THF formation at a rate of 680 nmol·min^−1^ mg^−1^ MtqA (n = 2). This rate is approximately 17% of the expected rate of MtyB methylation of Co(I)-MtqC and suggests MtqA was the rate-limiting enzyme in this assay. No detectable methyl-THF formation was observed in duplicate controls in which MtyB, MtqC, MtqA, or γ-butyrobetaine was withheld from the reaction.

## Discussion

Phylogenetic analysis indicates substantial diversity among the many MttB superfamily members encoded in the genome of *E. limosum* (Fig. S7), which led us to investigate their functional diversity (5, 6, 7). Here, we have shown that even two closely related nonpyrrolysine MttB homologs in the *E. limosum* genome have modified functionality in such a way as to allow their specialization for use with two distinct but related substrates. MtyB, a close relative of the l-carnitine methyltransferase MtcB (Fig. S7), is the first enzyme found, to our knowledge, to robustly catalyze the demethylation of γ-butyrobetaine.

MtyB is abundant in cells grown with γ-butyrobetaine. Previously, MtcB, the l-carnitine methyltransferase found in cells grown on l-carnitine, was shown to have detectable activity with γ-butyrobetaine (9). However, MtcB itself is undetectable in the proteome of γ-butyrobetaine-grown cells (Table S2). Indeed, no other MttB superfamily members than MtyB are abundant in these cells, and only MtpB, the proline betaine methyltransferase, was detectable at a very low abundance of 0.013 mol% protein. MtyB was not detectable in the proteome of *E. limosum* grown with l-carnitine or proline betaine (8, 9). Despite the observed *in vitro* catalytic promiscuity of MtyB for both γ-butyrobetaine and l-carnitine, the current and past proteomic data suggest that MtyB is specifically induced by γ-butyrobetaine, leading us to conclude that the physiological role of MtyB in *E. limosum* is that of a major butyrobetaine methyltransferase.

It is notable that MtyB can catalyze both γ-butyrobetaine:MtqC and l-carnitine:MtqC methyl transfer with comparable catalytic efficiency, whereas MtcB was found to much prefer l-carnitine as a substrate relative to γ-butyrobetaine. MtcB has much lower catalytic efficiency with γ-butyrobetaine than does MtyB. The ability of either MtyB and MtcB to utilize both γ-butyrobetaine and l-carnitine as methyl donors no doubt reflects the structural similarity between the two QAs, differing only in the presence of the β-hydroxyl group of l-carnitine and the unmodified β-methylene group of γ-butyrobetaine. It is tempting to speculate that MtcB residues interacting with the hydroxyl of l-carnitine further disfavor binding of the β-methylene of γ-butyrobetaine, necessitating the utilization of a discrete MttB superfamily member with similar catalytic efficiency for both QAs to utilize γ-butyrobetaine as the predominant growth substrate. Future structural studies may test this hypothesis. The catalytic efficiencies of MtyB and MtcB may also reflect the relative abundance of the two compounds in the colon. In human feces, γ-butyrobetaine can be found at low millimolar concentrations (16), whereas L-carnitine is found at much lower concentrations (49).

**Figure 6:**
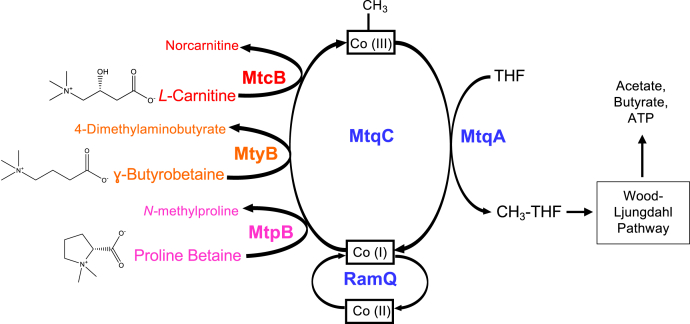
**Quaternary amine-dependent methylation of THF by *Eubacterium limosum*.** Our previous (8, 9) and current data support a model of quaternary amine metabolism in *E. limosum* in which MttB superfamily members have specialized for utilization of distinct substrates, such as MtpB, MtcB, and MtyB. Each superfamily member has retained recognition of a single cognate corrinoid-binding protein, Co(I)-MtqC, which further allows utilization of the same ATP-dependent reductive activation enzyme, RamQ, and the same methyl-Co(III)-MtqA:THF methyltransferase, MtqA to methylate THF with distinct quaternary amines. CH_3_-THF formed by demethylation of the quaternary amine can then directly enter the Wood–Ljungdahl pathway, where it is oxidized for reducing power and used directly in the synthesis of acetyl-CoA (63), which is used in the further synthesis of catabolic products acetate and butyrate (9, 64). THF, tetrahydrofolate.

MtyB joins MtcB (9) and MtpB (8) as the third nonpyrrolysine MttB homolog from *E. limosum* found to methylate the same corrinoid-binding protein, MtqC, which is subsequently demethylated by MtqA to form methyl-THF (Fig. 6). As found here for γ-butyrobetaine-grown cells, the abundances of MtqC, MtqA, and the corrinoid activase RamQ are each significantly increased in *E. limosum* grown on l-carnitine (9) or proline betaine (8) relative to cells grown on dl-lactate. In the *E. limosum* genome, MtqA and RamQ are encoded in a cluster of genes encoding enzymes that carry out the oxidation of methyl-THF, an essential pathway generating reducing power for subsequent acetogenesis. MtqC is encoded in a separate gene cluster with another corrinoid protein (8). We hypothesize the discrete encoding in separate gene clusters of the different QA methyltransferases, MtqC, and MtqA/RamQ may be of some benefit to the organism. The *mtqC*, *ramQ*, and *mtqA* genes may be inducible by any number of different QAs, whereas expression of genes encoding MttB superfamily members with distinct substrate preferences (*i.e.*, MtpB, MtcB, and MtyB) is induced only by their specific physiological QA (proline betaine, l-carnitine, and γ-butyrobetaine, respectively). Such a methyltransferase system may lend the organism a competitive advantage in the gut, allowing rapid response to quickly changing combinations of distinct dietary QAs.

Past studies have highlighted *E. limosum* as a microbe associated with good health, with various perceived benefits attributed to the production of short-chain fatty acids (50) and/or phytoestrogens (51, 52). Indeed, a large cohort microbiome study found correlation of *E. limosum* and closely related species with longevity. These bacteria were found to be 10-fold more abundant in centenarians (53). Given our current findings, this could be due to the possible cardioprotective advantage *E. limosum* gives its human host. Microbially produced γ-butyrobetaine is a major intermediate in the microbial production of TMA from dietary l-carnitine, and TMA is the major precursor to proatherogenic TMAO (15). Employing MtyB and MtcB, *E. limosum* can demethylate both these TMA precursors. To our knowledge, the fate of the demethylated products in the gut, that is, 4-dimethylaminobutyrate and norcarnitine, is unknown, but TMA production from either compound seems unlikely. However, the consumption of γ-butyrobetaine by MtyB and carnitine by MtcB might decrease TMA production in the gut *via* competition for these TMA precursors. The presence of microbes employing these enzymes thus may provide a natural means to control net TMA production by the gut microbiota. Future functional characterization of the numerous MttB homologs encoded by *E. limosum* may provide further insight into the perceived benefits of this organism for human health.

## Experimental procedures

### Growth of *E. limosum* by γ-butyrobetaine demethylation

*Eubacteriumlimosum* ATCC 8486 was cultured at 37 °C in the defined anaerobic low salt medium as described in the study by Kountz *et al.* (9), except for the following changes per liter: Na_2_SeO_3_•5H_2_O, 3 μg; Na_2_WO_4_•2H_2_O, 4 μg; 0.0001% resazurin; and NaHCO_3_, 1.8 g. Stocks of γ-butyrobetaine ((3-carboxylpropyl)trimethylammonium chloride; CAS 6249-56-5; Sigma–Aldrich) were adjusted to pH 7 with NaOH prior filtering through a Corning syringe filer (0.22 μm). The concentration of stock solutions was confirmed by quantitative NMR as described later.

Growth of *E. limosum* on 30 mM γ-butyrobetaine in 18 × 150 mm anaerobic tubes (Bellco Glass, Inc) under a 100% nitrogen atmosphere was monitored using a Spectronic 21 spectrophotometer. At each time point, 0.1 ml of each culture was anoxically removed and frozen at −80 °C for later analysis. Thawed samples were spun at 16,200*g* for 10 min to remove cellular debris, and the supernatant was analyzed by quantitative ^1^H-NMR. Each sample was added to an NMR tube and diluted 1:10 with 1 mM sodium trimethylsilylpropanesulfonate in 10% deuterium oxide to serve as the internal reference. Uninoculated media with γ-butyrobetaine or 4-dimethylaminobutyrate were similarly treated (Fig. S1) to verify peak assignment. ^1^H-NMR spectra were obtained at 298 K using an AVANCE III HD spectrometer (Bruker BioSpin) at a frequency of 800 MHz over a width of 20 ppm with 64 scans and a 3.53 min of acquisition time. ^13^C NMR spectra were also acquired using the same instrument with 128 scans with a 19 min acquisition time to provide further confirmation of the results from ^1^H-NMR. Baseline and phase corrections were applied to each spectrum using Top Spin 3.6.1 (Bruker). The proton signal from the three methyl groups of γ-butyrobetaine was located at 3.12 ppm (^1^H) and 55.56 ppm (^13^C). The proton signal from the two methyl groups of 4-dimethylaminobutyrate was located at 2.88 ppm (^1^H) and 45.38 ppm (^13^C).

### Cloning, expression, and purification of recombinant proteins

MtyB and MtqC were coexpressed in *E. coli* Tuner. A codon-optimized version of the *E. limosum mtyB* gene sequence (Fig. S3) was produced using COOL (54) and synthesized by Life Technologies/Thermo Fisher Scientific. Upon delivery, the codon-optimized *mtyB* gene was digested with NdeI and XhoI and ligated with T4 ligase into pET-26b(+). To generate the pETDuet-1 coexpression vector, the *mtyB* gene was transferred as a NdeI/XhoI fragment from pET-26b(+) into multiple cloning site 2 of pETDuet-1 using T4 ligase. The *mtqC* gene bearing a C-terminal Strep II tag was amplified from the previously constructed pSpeedET expression vector (8) using *mtqC* forward and reverse primers (Table S5). The PCR product was digested with NcoI/BamHI and then ligated into multiple cloning site 1 of the MtyB/pETDuet-1 clone. Finally, the C-terminal S•Tag conferred onto MtyB by multiple cloning site 2 was converted to a C-terminal hexahistidine tag using the mtyB-His forward and reverse primers (Table S5). The *mtyB* and *mtqC* genes were then coexpressed in *E. coli* Tuner grown in LB medium containing 100 μg/ml ampicillin. Recombinant protein production was induced when the culture reached 0.4 at an absorbance at 600 nm with 0.25 mM IPTG followed by 2.5 h incubation with shaking at room temperature prior to centrifugation. The cell pellets from 3 l culture were flash frozen in liquid nitrogen and stored at −80 °C. To purify recombinant MtyB, the cell pellet was thawed and resuspended in buffer A (10% glycerol, 20 mM imidazole, and 500 mM NaCl in 20 mM sodium phosphate, pH 7.2). A few crystals of DNAase were added, and the cells were lysed using a French pressure cell. The lysate was centrifuged at 41,000*g* at 4 °C for 45 min. The clarified lysate was then loaded onto two tandem 1-ml His-Trap columns (GE), and the columns were eluted with a 40 ml linear gradient of 20 to 500 mM imidazole in buffer A. The hexahistidine-tagged MtyB coeluted with Strep II–tagged MtqC in a broad peak between 58 and 173 mM imidazole (Fig. S4). MtyB was resolved from MtqC using DE-52 (Whatman). MtyB-containing fractions from the His-Trap were pooled, then diluted 10-fold into 50 mM NaCl and 10% glycerol in 50 mM Tris, pH 8, and loaded onto a 30 ml DE-52 column. The column was then eluted with 150 ml of a linear gradient of 50 mM to 1 M NaCl in the same buffer. Purified MtyB (Fig. S5) eluted between 250 and 354 mM NaCl. A second peak of coeluting MtyB and MtqC was noted between 430 and 544 mM NaCl but was discarded.

Recombinant MtcB was obtained using the previously described expression plasmid (9), but with a different purification protocol. The *mtcB* gene was induced in *E. coli* BL21 growing in 4 l of LB medium containing 50 μg/ml kanamycin sulfate with 1 mM IPTG when the culture reached at an absorbance of 0.4 at 600 nm. The culture was then incubated with shaking at room temperature for 2.5 h prior to collecting cell pellets by centrifugation, which were then frozen with liquid nitrogen and stored at −80 °C until purified. The pellet was thawed and resuspended in buffer A, then lysed, clarified, and purified by nickel affinity chromatography as described previously. MtcB eluted in a broad peak between 68 and 154 mM imidazole. Nickel-purified fractions were pooled and diluted 10-fold in 10% glycerol and 50 mM NaCl in 20 mM Tris buffer, pH 7.5, prior to purification employing a DE-52 column as described previously. MtcB eluted between 211 and 259 mM NaCl. The protein-containing fractions were pooled and then concentrated to 300 μl using Amicon Ultra-0.5 ml spin columns. The concentrated protein was then applied to a Superose 12 10/300GL column, and the purified protein was eluted with 150 mM NaCl and 10% glycerol in 50 mM sodium phosphate, pH 7.5.

Strep II-tagged MtqC was produced per the protocol published by Picking *et al.* (8). Briefly, the *mtqC* gene was expressed in *E. coli* BL21 aerobically, in LB medium containing 50 μg/ml kanamycin sulfate. Clarified cell lysates were made in 1 mM EDTA and 300 mM NaCl in 100 mM Tris, pH 8.0. Then loaded onto a 5 ml Strep-Tactin XT high-capacity column (IBA Life Sciences), and the column was washed with the same buffer prior to elution with 50 mM biotin in the same buffer. Apo-MtqC was stirred anaerobically overnight in 3.5 M betaine and 1 mM hydroxocobalamin in 50 mM Tris–HCl, pH 7.2. The concentrated MtqC holoprotein was then applied to a 23.6 ml Superose 12 10/300GL column equilibrated with 150 mM NaCl and 10% glycerol in 50 mM Tris, pH 7.2, and eluted with the same buffer in order to further purify the protein and remove nonprotein-bound hydroxocobalamin (Fig. S5). Preparations of MtqC used in these experiments possessed an average of 0.7 mol cobalamin/mol protein.

The *mtqA* gene was expressed in *E. coli* BL21 in LB medium containing 50 μg/ml kanamycin sulfate as previously described (8). The *mtqA* gene was induced at room temperature with 1 mM IPTG at a culture of 0.4 at an absorbance at 600 nm, and the culture was shaken for 2.5 h prior to harvesting. The combined cell pellets were flash frozen with liquid nitrogen and stored at 80 °C. To purify the hexahistidine-tagged MtqA, the pellet was thawed and resuspended in 10% glycerol, 20 mM imidazole, 500 mM NaCl in 20 mM sodium phosphate, pH 7.2. The cells were then lysed and clarified by centrifugation prior to MtqA purification using two tandem 1 ml Hi-Trap columns (GE) as described previously. MtqA eluted in a broad peak between 68 and 193 mM imidazole.

Recombinant RamQ was produced and purified following the previously described anoxic protocol for ATP-dependent corrinoid protein reductases (55). All purification steps were performed anaerobically, and column chromatography conducted inside a Coy anaerobic chamber. The *ramQ* gene was expressed in *E. coli* SG13009 under a nitrogen atmosphere in anaerobic LB medium containing 80 mM d-glucose, 80 mM sodium fumarate, 30 μg/ml kanamycin sulfate, and 44 mM potassium phosphate buffer at pH 7.2. The *ramQ* gene was induced with 1 mM IPTG at an absorbance of 0.4 at 600 nm, at which time 0.1 mM ferrous ammonium sulfate and 1 mM cysteine–HCl were added to the culture. After 2 h, 0.1 mM ferrous ammonium sulfate and 1 mM cysteine–HCl were again added to the culture. After two more hours, the cell pellet was harvested anaerobically and stored at −80 °C. To purify RamQ, the pellet was thawed and resuspended in anaerobic 20 mM imidazole, 2 mM DTT, and 500 mM NaCl in 20 mM sodium phosphate at pH 7.2. A few crystals of DNAase were added, and the cells were passed through a French press and collected in an anaerobic tube. The lysate was centrifuged at 41,000*g* for 45 min at 4 °C. The clarified lysate was then loaded onto a 1 ml His-Trap (GE), washed with buffer A, and then eluted with a 40 ml gradient from 100% A to 100% buffer B (buffer A with 500 mM imidazole). RamQ eluted between 150 and 212 mM imidazole. RamQ was then loaded onto an 8 ml MonoQ column and eluted over a 40 ml linear gradient from 0 to 500 mM NaCl (buffer A: 50 mM MOPS, pH 7; buffer B: 1 M NaCl in 50 mM MOPS, pH 7). RamQ eluted between 200 and 240 mM NaCl.

### Spectrophotometric MtqC methylation assays with MtyB or MtcB

The formation of methyl-Co(III)-MtqC from Co(I)-MtqC was monitored spectrophotometrically in anoxic stoppered quartz submicro cuvettes (Starna Cells, Inc) with a path length of 1 cm. Reaction rates were calculated using either the increasing absorbance at 534 nm or the decreasing absorbance at 386 nm. Isosbestic points at 578 and 439 nm were also followed to assure that the Co(I) form of MtqC was converted to the methylated form without appreciable accumulation of Co(II)-MtqC. Extinction coefficients for methyl-Co(III)-MtqC and Co(I)-MtqC calculated by Picking *et al.* (8) were used to quantify the concentration of each form of MtqC over the course of assays. Reactions were performed at 37 °C under dim red light to avoid photolysis of the methylated cobalamin cofactor. The assay cuvettes were prepared and stoppered inside an anaerobic Coy chamber with an atmosphere of 2% H_2_:98% N_2_. Basal reaction mixtures included 2 mM Ti(III)citrate, 2.5 mM MgCl_2_, 2.5 mM ATP, 100 mM potassium acetate, and either γ-butyrobetaine or l-carnitine in 22 mM potassium phosphate buffer at pH 7.2. All proteins were added to the stoppered cuvettes outside the anaerobic chamber using flushed Hamilton syringes. UV–visible spectra of the reaction were sampled every second using an HP 8453 Diode-Array Biochemical Analysis Spectrophotometer. Each reaction had a final volume of 100 μl. Stoppered cuvettes with the basal reaction mixture were used to blank the spectrophotometer. MtqC was then added, followed by RamQ. The enzymatic reduction of added Co(II)-MtqC to Co(I)-MtqC was monitored by the loss of the characteristic Co(II) peak at 475 nm and the appearance of the representative Co(I) peak at 386 nm. Methylation was then initiated by addition of either MtyB or MtcB. Assays to determine the apparent *K*_*m*_ of MtyB for γ-butyrobetaine included 240 nM recombinant MtyB, 5 μM RamQ, and 30 μM MtqC. Assays to determine the *K*_*m*_ of MtyB for l-carnitine included 80 nM recombinant MtyB, 5 μM RamQ, and 15 μM MtqC. Tested l-carnitine or γ-butyrobetaine concentrations were 0.5, 1, 2, 5, 10, and 20 mM. Assays to determine the *K*_*m*_ of MtcB for γ-butyrobetaine included 1.1 μM recombinant MtcB, 4.5 μM RamQ, and 56 μM MtqC. Tested γ-butyrobetaine concentrations were 5, 10, 15, 20, and 50 mM. Assays with MtyB with all other substrates were performed using 2 μM MtyB, 4.5 μM RamQ, 30 μM MtqC, and 25 mM substrate.

### Assay of γ-butyrobetaine:THF methyl transfer

Reactions were performed under dim red light in a Coy anaerobic chamber under an atmosphere of 2% H_2_:98% N_2_. Reaction mixtures (200 μl) contained 2 μM MtyB, 60 μM MtqC, 1.2 μM MtqA, 4.5 μM RamQ, 5 mM γ-butyrobetaine, 1.25 mM ATP, 1.25 mM MgCl_2_, 2 mM Ti(III) citrate in 22 mM potassium phosphate buffer, and pH 7.2. Reaction vials were preincubated at 37 °C for 5 min prior to initiation by the addition of 4.5 mM THF. Aliquots (30 μl) were removed at the indicated time points and quenched with 6 μl saturated trichloroacetic acid. The reaction time points were centrifuged to remove precipitated protein and stored anaerobically in the dark at −80 °C until analysis at which time samples were injected into a 250 × 4.6 mm Varian Microsorb MV-100 C18 column attached to a Dionex UltiMate 3000 HPLC system. The column was then eluted with 7% (v/v) acetonitrile in 30 mM potassium phosphate buffer, pH 3.0 at 0.5 ml/min. THF and methyl-THF were detected at 272 nm with elution times of 15.5 and 20 min, respectively. Peak integration was performed using Chromeleon 6.8 (Dionex), and methyl-THF was quantified using a standard curve.

### Phylogenetic tree of MttB homologs from *E. limosum*

The *Desulfitobacterium hafniense* glycine betaine methyltransferase MtgB (UniProtKB—Q24SP7) (5) was used as a query for a BLASTP (56) search of the genome of *E. limosum* ATCC 8486 (GenBank ID: CP019962.1 (45) at the National Institute of Biotechnology site). The sequences of 42 nonpyrrolysine MttB homologs were retrieved and combined with the sequences of the MtgB homolog from *Acetobacterium woodii* (UniProtKB—H6LKF8) (7), MttB from *Methanosarcina barkeri* (UniProtKB—O93658) (4), and MV8460 from *Methanolobus vulcani* Bd1 (6). The amino acid sequences were aligned using MUSCLE (57) in Seaview (58). Phylogenetic analysis was then performed using maximum likelihood with MEGA (59), using the JTT model (60) and 1000 bootstrap replicates. The MEGA-generated tree was uploaded to iTOL, where it was visually modified for publication (61).

### Proteomic analysis of *E. limosum*

The proteome of cells grown on γ-butyrobetaine was analyzed using the previously described methodology used to determine the proteome of cells grown on proline betaine, l-carnitine, or dl-lactate (8, 9). Four independent 10 ml *E. limosum* cultures were grown on the aforementioned LS medium supplemented with 50 mM γ-butyrobetaine. Each culture was anaerobically harvested between an absorbance of 0.50 to 0.55 at 600 nm, and the cell pellet was washed once with 22 mM potassium phosphate buffer prior to freezing. The proteome from each cell pellet was subsequently individually analyzed to yield four biological replicates. From each frozen cell pellet, an aliquot was lyophilized, subjected to cryogrinding, protein extraction, reduction with DTT and alkylation with iodoacetamide as described previously (9) prior to proteolysis with sequencing grade–modified trypsin (Promega). This grade of trypsin is devoid of chymotrypsin activity and cleaves at the carboxylic side of lysine or arginine. The peptide mixtures (6 μg) from each of the four cell pellets were then subjected to online 2D LC separation using Thermo Fisher Scientific 2D RSLC HPLC system. The sample was first fractionated on a 5 mm × 300 μm BEH C18 column with 3 μm particle size and 130 Å pore size. Solvent A was composed of 20 mM ammonium formate, pH 10, and solvent B was composed of 100% acetonitrile. Peptides were eluted from column in eight successive fractions using 9.5, 12.4, 14.3, 16.0, 17.8, 19.7, 22.6, and 50% solvent B. Each eluted fraction was then online trapped, neutralized, and desalted on a μ-Precolumn Cartridge (Thermo Fisher Scientific) for the 2D separations performed on a 15 cm × 75 μm PepMap C18 column (Thermo Fisher Scientific) with 3 μm particle size and 100 Å pore size. Mobile phase A was 0.1% formic acid in water, and mobile phase B was 0.1% formic acid in acetonitrile, and a flow rate of 500 μl/min was used. The gradient was from 0 to 5 min, 3% solvent B; from 5 to 35 min, 5 to 35% solvent B; from 35 to 42.5 min, 35 to 55% solvent B; and for 42.5 to 44.5 min, 55 to 85% solvent B. Solvent B was then kept at 85% for 1 min before being returned to 3% solvent B. The system was equilibrated for 14.5 min before the next separation. MS/MS data were acquired with a spray voltage of 1.7 kV and a capillary temperature of 275 °C. The scan sequence of the mass spectrometer was based on the preview mode data-dependent TopSpeed method: the analysis was programmed for a full scan recorded between *m/z* 400 and 1600 and a MS/MS scan to generate product ion spectra to determine amino acid sequence in consecutive scans starting from the most abundant peaks in the spectrum in the next 3 s. To achieve high mass accuracy MS determination, the full scan was performed at FT mode and the resolution was set at 120,000. The automatic gain control target ion number for FT full scan was set at 2 × 10^5^ ions, maximum ion injection time was set at 50 ms, and micro scan number was set at 1. MS/MS was performed using ion trap mode to ensure the highest signal intensity of MS^2^ spectra using both collision-induced dissociation (for 2+ and 3+ charges) and ETD (for 4+ to 7+ charges) methods. The automatic gain control target ion number for ion trap MS^2^ scan was set at 1000 ions, maximum ion injection time was set at 100 ms, and micro scan number was set at 1. The collision-induced dissociation fragmentation energy was set to 35%. Dynamic exclusion is enabled with a repeat count of 1 within 60 s and a low mass width and high mass width of 10 ppm. Sequence information from the MS/MS data was processed by converting raw files into mgf files using MSConvert, version 3.0.8738 (ProteoWizard), and then mgf files from each of the fractions were merged into a single merged file (mgf) using an in-house program, RAW2MZXML_n_MGF_batch (merge.pl, a Perl script). The resulting mgf files were searched using Mascot Daemon (version 2.5.1) by Matrix Science against the set of 3998 proteins encoded in genome of *E. limosum* ATTC 8486 (45) (GenBank assembly accession: GCA_000807675.2, deposited on March 4, 2017) and maintained at the National Center for Biotechnology Information. The mass accuracy of the precursor ions was set to 10 ppm, and an allowance for selection of one ^13^C peak for each identified peptide was also included into the search. The fragment mass tolerance was set to 0.5 Da. Considered variable modifications were oxidation (Met), deamidation (Asn and Gln), and carbamidomethylation (Cys). Four missed cleavages for the enzyme were permitted. A decoy database was also searched to determine the false discovery rate (FDR), and peptides were filtered according to the FDR. The significance threshold for peptide identification was set at *p* < 0.05. Only proteins identified with <1% FDR as well as a minimal of two unique peptides are accepted for quantitation. Label-free quantitation was performed using the spectral count approach, in which the relative protein quantitation is measured by comparing the number of MS/MS spectra identified from the same protein in each of the multiple LC/MS–MS datasets. Scaffold (Proteomic Software, Inc) was used for data analysis and calculation of exponentially modified protein abundance index values to estimate the mol% of each identified protein within the total set of identified proteins (47). Standard deviations were calculated of the estimated molar abundance of each identified protein in the four biological replicates. Student's *t* test was performed using Scaffold to evaluate if the fold change for certain proteins between growth conditions is significant (*p* < 0.05).

## Data availability

The MS proteomics data have been deposited to the ProteomeXchange Consortium *via* the PRIDE partner repository (62) with the project accession number PXD013806 and project DOI 10.6019/PXD013806 (for the previously obtained lactate dataset (8)) and with the identifier PXD020152 and DOI 10.6019/PXD020152 (for the γ-butyrobetaine dataset). All other discussed data can be found in the article, supporting information files, and cited references.

## Supporting information

This article contains supporting information (8, 9, 45, 59).

## Conflict of interest

The authors declare that they have no conflicts of interest with the contents of this article.
