# Supplementary material for: The MttB superfamily member MtyB from the human gut symbiont Eubacterium limosum is a cobalamin-dependent γ-butyrobetaine methyltransferase
Source: J Biol Chem. 2021 Oct 21;297(5):101327. doi: 10.1016/j.jbc.2021.101327 (PMC8604678; doi:10.1016/j.jbc.2021.101327)
Supplement: Supplemental Figures S1–S7 and Tables S3–S5 [file mmc3.pdf]

Supporting Information

The MttB superfamily member MtyB is a cobalamin-dependent γ-butyrobetaine methyltransferase from the human gut symbiont *Eubacterium limosum*

Jared B. Ellenbogen[1], Ruisheng Jiang[1], Duncan J. Kountz[1#], Liwen Zhang[2], and Joseph A. Krzycki[1,3*]

From the: [1]Department of Microbiology, [2]Campus Chemical Instrument Center Mass Spectrometry and Proteomics Facility, [3]The Ohio State Biochemistry Program, The Ohio State University, Columbus, OH 43210

Running Title: *Methylation of tetrahydrofolate with γ-butyrobetaine*

[#]Present address: Department of Chemistry and Chemical Biology, Harvard University, Cambridge, MA 02138

*To whom correspondence should be addressed: Joseph A. Krzycki: Department of Microbiology, The Ohio State University, Columbus OH 43210; Krzycki.1@osu.edu; (614) 292-1578; Fax. (614)292-8120.

This PDF file includes:

Tables S3 to S5

Figs. S1 to S7

Other supplementary files for this manuscript include the following:

Dataset S1(Table S1)

Dataset S2 (Table S2)

Table S3. Proteins of acetogenesis in γ-butyrobetaine and *DL*-lactate grown cells.

**Name**	**Accession number**	**Predicted Function**	**Percent of total soluble protein observed in γ-butyrobetaine grown cells**	**Percent of total soluble protein observed in lactate grown cells**	**Fold change***	**P-value**
**FdhA**	WP_038354071.1	Formate dehydrogenase subunit α	0.047± 0.024	0.027 ± 0.0069	1.7	0.17
**Fhs1**	WP_038351869.1	Formate-THF ligase	0.31 ± 0.13	0.16 ± 0.032	1.9	0.07
**FchA**	WP_038351868.1	Methenyl-THF cyclohydrolase	0.054 ± 0.012	0.024 ± 0.0080	2.3	0.01
**FolD**	WP_038351867.1	Methylene-THF dehydrogenase	0.23 ± 0.071	0.12 ± 0.014	1.9	0.03
**MetV**	WP_038351866.1	5,10-Methylene-THF reductase	1.5 ± 0.52	1.5 ± 1.1	1.0	0.97
**MetF**	WP_081571099.1	5,10-Methylene-THF reductase	0.11 ± 0.050	0.081 ± 0.013	1.4	0.28
**AcsE**	WP_013381869.1	Carbon monoxide dehydrogenase	5.2 ± 0.47	6.3 ± 1.6	0.83	0.23
**AscC**	WP_038352891.1	Acetyl-CoA synthase subunit γ	0.59 ± 0.24	0.88 ± 0.56	0.67	0.38
**AscD**	WP_038352892.1	Acetyl-CoA synthase subunit δ	2.7 ± 1.5	2.4 ± 0.46	1.1	0.74
**AscA**	WP_038352890.1	Carbon-monoxide dehydrogenase catalytic subunit	0.24 ± 0.062	0.14 ± 0.01 1	1.7	0.02
**AscB**	WP_038352888.1	Bifunctional acetyl-CoA decarbonylase/synthase complex subunit α/β	0.12 ± 0.051	0.051 ± 0.013	2.4	0.03

*Ratio of mol % protein in γ-butyrobetaine- versus *DL*-lactate-grown cells.

Table S4. Proteins of lactate catabolism in *DL*-lactate and γ-butyrobetaine grown cells.

Name	Accession number	Predicted Function	Percent of total soluble protein observed in γ-butyrobetaine grown cells	Percent of total soluble protein observed in lactate grown cells	Fold change*	P-value
LctA	WP_052237246.1	Transcriptional regulator	0.0008 ± 0.0002	0.0034 ± 0.0017	0.24	0.015
LctB	WP_038352354.1	Electron transfer flavoprotein subunit β	Not detected	2.0 ± 0.89	≤0.00005[A]	0.0039
LctC	WP_038352355.1	Electron transfer flavoprotein subunit α	Not detected	2.6 ± 0.96	≤0.00004[A]	0.0017
LctD	WP_013380257.1	Lactate dehydrogenase	Not detected	1.9 ± 0.52	≤0.00005[A]	0.00034
LctE	WP_038352356.1	Lactate permease	Not detected	0.003 ± 0.00087	≤0.03[A]	0.00042
LctF	WP_038352908.1	Lactate racemase	0.0018 ± 0.00092	0.23 ± 0.097	0.0078	0.0034
PorA	WP_038354167.1	Pyruvate:ferredoxin oxidoreductase	0.097 ± .037	2.6 ± 1.2	0.04	0.01

*Ratio of mol % protein in γ-butyrobetaine- versus *DL*-lactate-grown cells.

[A]*a lower limit of detection of 0.0001% of total soluble protein was used to estimate this value.*

Table S5. Primers utilized in this work.

Primer	Sequence
PIPE forward insert	AATTAAGTCGCGTTATTATTTGATCTTGAATTTTTCCGGAAGGTG
PIPE reverse insert	AATTAAGTCGCGTTATTATTTGATCTTGAATTTTTCCGGAAGGTG
PIPE forward vector	GCCCTGGAAGTACAGGTTTTCGTGATGATGATGATGATG
PIPE reverse vector	TAACGCGACTTAATTAAACGGTCTCCAGCTTGGCTGTTTTGGC
mtqC forward	TATACCATGGCAGATTGGAAAAATTTAACACAGG
mtqC reverse	TCTCGGATCCTCACTTTTCAAATTGTGG
mtyB-His forward	ATGGTGATGGTGATGTCCTCCGCCTTTAATTTTAAATTTTTCCGGCAG
mtyB-His reverse	CATCACCATCACCATCACTGACTCGAGTCTGGTAAAGAAACCGCTG

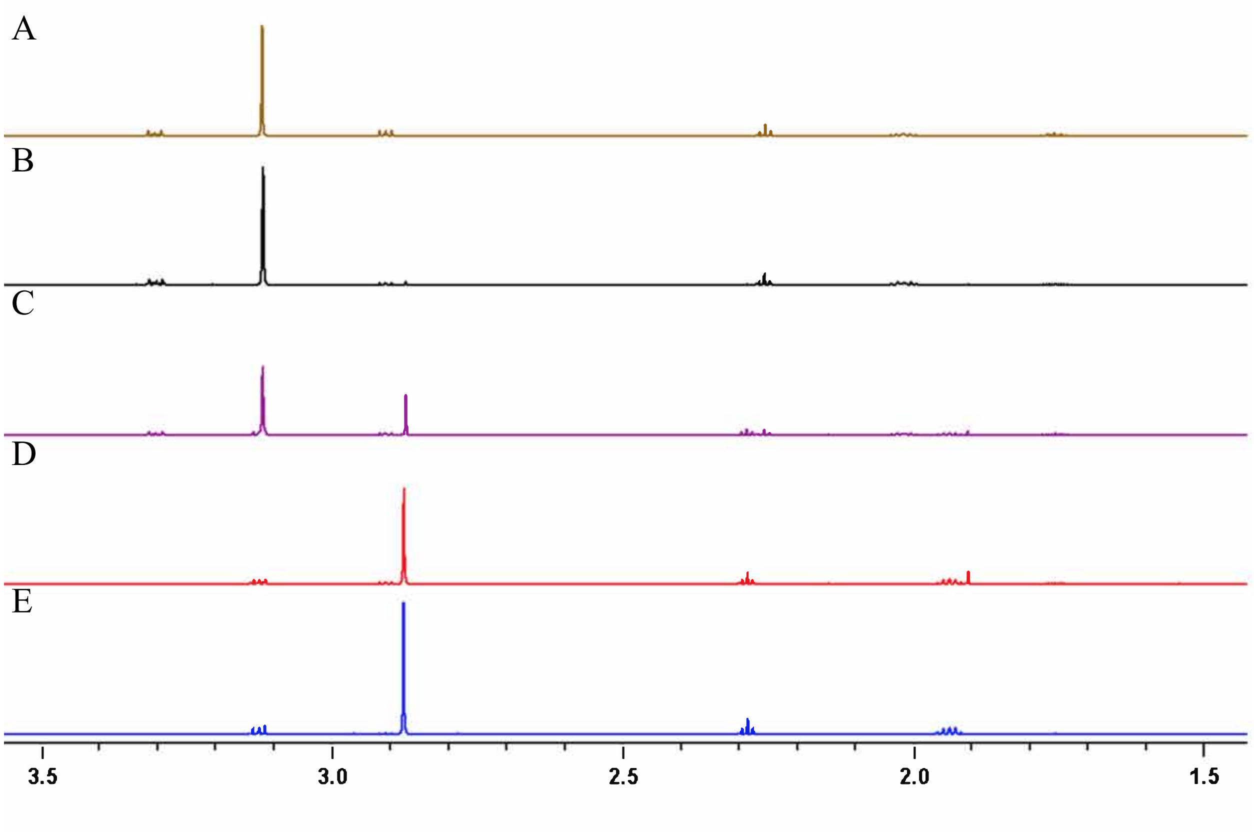

Figure S1. *E. limosum* demethylates γ-butyrobetaine to produce 4-dimethylaminobutyrate. Samples of culture supernatant removed before, during and after growth were subjected to quantitative ^{1}H-NMR in order to track conversion of γ-butyrobetaine to the singly demethylated product. Each sample was diluted 1:10 in 10% D_2O containing 1 mM sodium trimethylsilylpropanesulfonate to serve as an internal reference. Integration of the $(CH_3)_3$-N- peak at 3.12 ppm of γ-butyrobetaine, and the $(CH_3)_2$- N-peak at 2.88 ppm of 4-diamethylminobutyrate was used to calculate consumption of the quaternary amine and production of the tertiary amine in Fig. 1B. The spectra were obtained with the following samples: (A) uninoculated medium containing 30 mM γ-butyrobetaine; (B) culture supernatant at inoculation (0 hrs); (C) culture supernatant after 119 hrs; (D) culture supernatant after 175 hrs; and (E) uninoculated medium containing 30 mM 4-dimethylaminobutyrate.

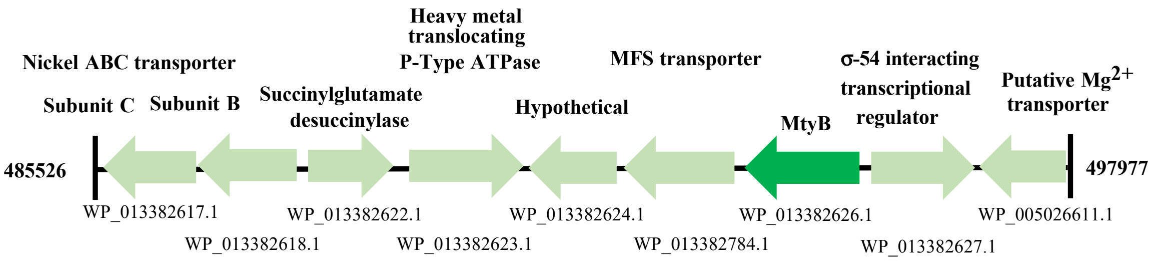

Figure S2. Genomic context of *mtyB* within the *E. limosum* ATCC 8486 genome (45). Similar to the *mtcB* (9) and *mtpB* (8) genes encoding the carnitine and proline betaine methyltransferases, the *mtyB* gene encoding the butyrobetaine methyltransferase is not found near the *mtqA*, *mtqC*, or *ramQ* genes encoding the other components of the THF methylation system. The genomic context of the latter genes was discussed previously (8). In brief, *mtqA* is found next to *ramQ*, whereas *mtqC* is next to a suite of genes encoding the enzymes that interconvert the various oxidation states of the one carbon unit bound to THF.

ATGCTGAAGGGTAACCTGTTTACGACTTTTTACTCTAAAGACGACATTGAACTGATTCATGAGAGTGTACTG
CGTGTTTTTAACGAGGTCGGTGTGAAGTTCGAGTATGAAGAAGCTCTGGAGTTGTTCAAAAAACACGGCGC
TCGCGTTGAAGGTGATCTGGTGTTTATCCCGGAAGAGTTGCTGAATAAGGCATTGTCCACTATTCCAGAAA
GCTTTGAGATCGTTGGGCCAGAGAGCAAATTGGAAATTGGCATCGGTAAGGGCTTGGTTGTTGCTCCGAC
CAATGGCTGTCCAAATCTGGAAGACTGGGATGGTAATTACCGCCAAACCAATGCTGATGACTTGGAGAACT
TTTACAAGATTATTAATACCTCCGATGTTTATGGCATTTCGTCCCAGGTGGCGGCAGACATTCCAGGTTTCG
AAAATTGCCCTACGGAGTCGGCTTTGGCGCAAATGGCAATGATGGCAAAATATTCCCGCAAACCAATGTAC
AACATTCTGGGTATCACTCCGCATAACTACAAGCGCGGCAATGCCCGCGAAGGTGCCCGCGAGTGCGTCC
AACTGATTAAAAAGTACATGGACAATTTTGATGATTATGTGTGTTATAGTGGTATGTGTGTATTGCCACCGCT
GACCGTAGGCACTGATGCGCTGGAGCACTATTTCGCATTTGCGGAGGAAAAACAGCCGATTACCATTACCA
CGTGTTCTATGACTAACATGACCGCCCCGCCATCTCTGATGGGCTCTATTGTTGCCGATTTCGCGAACATG
CTGGCCGTTGCAGTTATGATCCAATTGATCGAGCCGGGCTTGCCTGTTATCCTGTCGCCATTCTCGTCTGT
ATCGGATATGCGTGAGGTGCGCTTGATGACTGGCGCGCCGGAATTCTTGATGATTATGAGCGGTCATATC
GCTATGGGCGATTTCTACCGTATCCCGGTCCGTTGCAGCGGCGCTTTGGCAGACGGCTTCACTTACGATTA
TCAAGCAGGTGTTGAGTCGACTCTGGGTGCTCTGGCTGTAAGTCTGAGTAACGCTTGTATCTTGCCGCATG
CCTCGGGTGATTTGAGTAACTTCAACTTGTTGTCTTTTCCGAAGTTCATGATGGACGAAGAAATGCTGCGTT
ATATGAAGCGCCTGCACGACGGCGTAGCGATTTCCGAAAAGAAAGCCAATGTGGAGTTGATTAAGAAGGT
CGGCCCGCGCGGTGTATACTTGAAAGGCCGCACGCCGAAGGACTACCGTGAGGAGACCTATTTGACTAGT
CCGGTATTTAACCGCGGCACTTGTAACGAGGAAGGTCGTGCAGCGACCCCGCCAATTCAAGAGCGCGCCT
ATAAGGTGTACAAAGAGCGCATCGAAAGCTTTGAATTGCCTGACATGACGCAAGCTCAGAAGGACTTACTG
AATGAGCACCTGCCAGAAAAGTTCAAAATTAAA

Figure S3. Codon optimized sequence of recombinant *mtyB* used in this study.

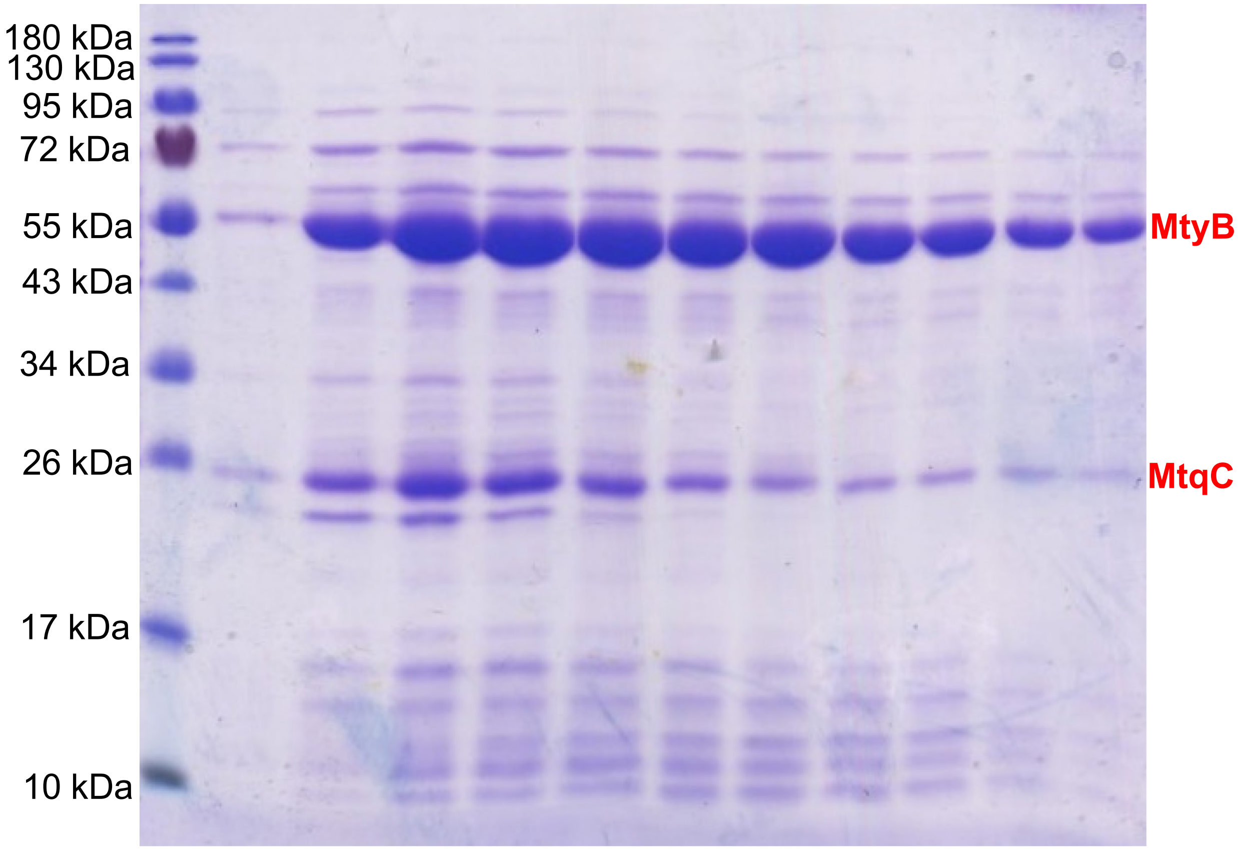

Figure S4. SDS-PAGE of His-trap (GE) fractions of semi-purified MtyB. A cell lysate of recombinant *E. coli* bearing genes encoding both MtyB (with a hexahistidine tag) and MtqC (with a Strep II tag) was loaded onto the nickel-affinity column. Following elution of unbound protein, a 23 kDa band corresponding to the mass of MtqC coeluted with hexahistidine-tagged MtyB (55 kDa) from the nickel column in a single peak. Molecular masses of standard proteins are indicated.

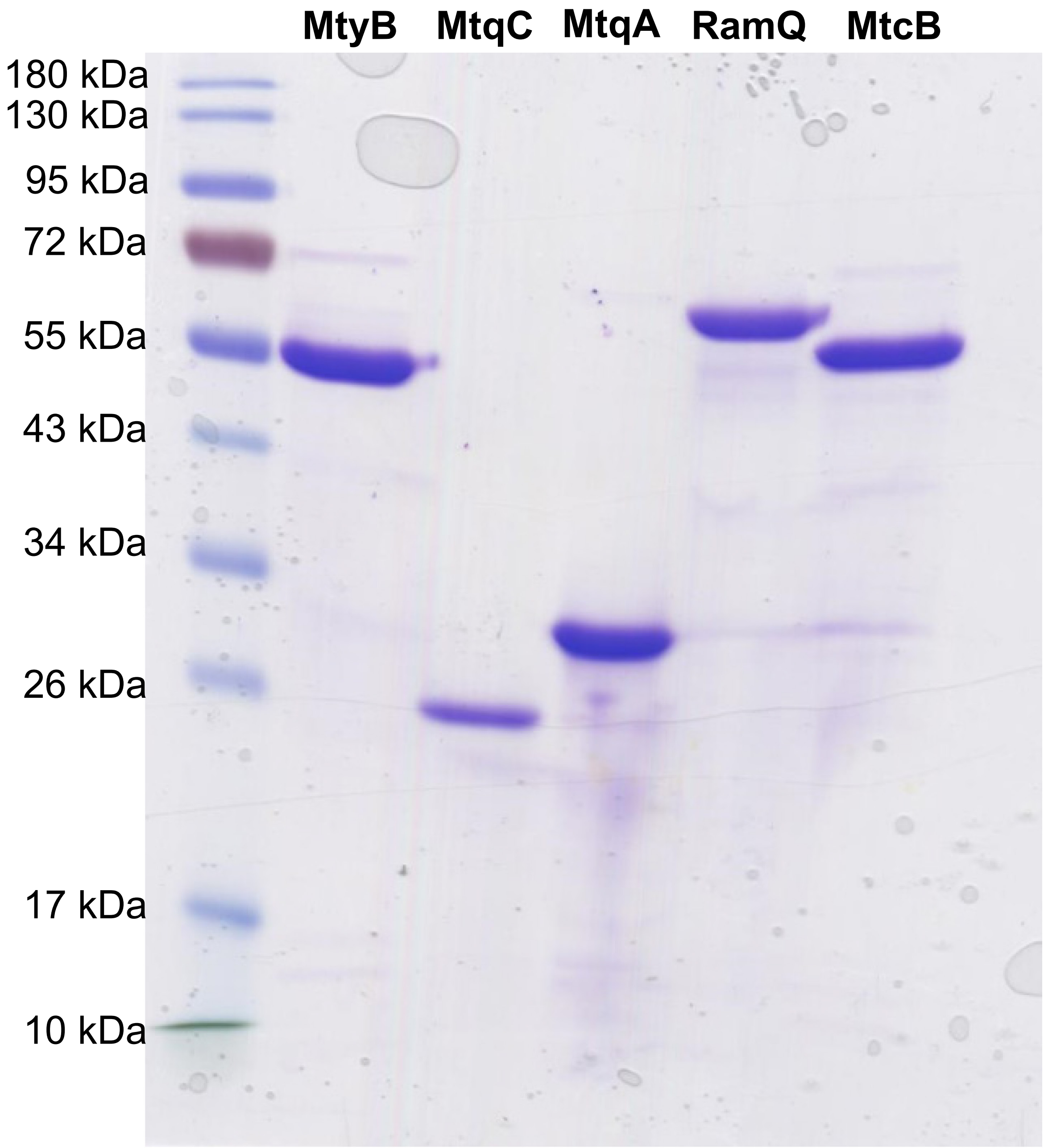

Figure S5. SDS-PAGE of the recombinant proteins (5 μg each) used in this study. For reference the predicted masses of each protein are as follows: MtyB, 55 kDa; MtqC, 23.4 kDa; MtqA, 32.1 kDa; RamQ, 66.7 kDa; MtcB, 56.4 kDa. Molecular masses of standard proteins are indicated.

```
MtyB  1    MLKGNLFTTFYSKDDIELIHESVLRVFNEVGVKFEYEEALELFKKHGARVEGDLVFIPEE  60
           M++ +L   F++KDD+E +HE VLRV ++VGVK E +EAL +F++HGARVE   V+I E
MtcB  1    MIRNSLTDVFFAKDDVENLHEGVLRVLSKVGVKIENDEALGIFEQHGARVENGTVYIGEV  60

MtyB  61   LLNKALSTIPESFEIVGPESKLEIGIGKGLVVAPTNGCPNLEDWDGNYRQTNADDLENFY  120
           LLNKAL T+P +FE+ G +   +++G+    VV PTNG P + ++DG+Y  TN DDL NFY
MtcB  61   LLNKALQTVPANFELQGFDRTVQVGLDHDPVVIPTNGTPMVLNFDGSYSDTNTDDLVNFY  120

MtyB  121  KIINTSDVYGISSQVAADIPGFENCPTESALAQMAMMAKYSRKPMYNILGITPHNYKRGN  180
           K+I+TSDV  ++S++A D+PG +    +S LAQ A++ KYS KP+YNILG T HNYK+G+
MtcB  121  KLIDTSDVMQVTSEIAVDVPGLDKTK-DSLLAQTALLMKYSHKPIYNILGATIHNYKKGS  179

MtyB  181  AREGARECVQLIKKYMDNFDDYVCYSGMCVLPPLTVGTDALEHYFAFAEEKQPITITTCS  240
             +G RE +Q  KKY    D YV YSG CV+ PL VG +A++H+  F +E QPI+IT CS
MtcB  180  VAQGVRENIQFAKKYYGYDDKYVIYSGTCVISPLGVGWEAMDHFMGFIKENQPISITACS  239

MtyB  241  MTNMTAPPSLMGSIVADFANMLAVAVMIQLIEPGLPVILSPFSSVSDMREVRLMTGAPEF  300
           MTN+TAP SL GS+V D A +L++ V+ QL+ PGLPV+ +  SS+SDMR V+L  GAPEF
MtcB  240  MTNLTAPGSLYGSVVEDAAAILSIVVLSQLMNPGLPVLYTSLSSMSDMRYVQLCMGAPEF  299

MtyB  301  LMIMSGHIAMGDFYRIPVRCSGALADGFTYDYQAGVESTLGALAVSLSNACILPHASGDL  360
            +I  GHIA+ +FY+IPVR  GAL D F  DYQAGVES +G +A  LS + ++PH  G +
MtcB  300  ALITLGHIALANFYKIPVRVGGALGDAFKADYQAGVESFVGLMAPMLSQSAMIPHGCGTM  359

MtyB  361  SNFNLLSFPKFMMDEEMLRYMKRLHDGVAISEKKANVEL--IKKVGPRGVYLKGRTPKDY  418
            +FNL S+ KF+MDEE +RY+ RL  G  +S+K+    L  I KVGPRG +L GRTPK+Y
MtcB  360  GSFNLTSYEKFIMDEETIRYLMRLRRGFEVSDKRKEKALKDITKVGPRGNFLGGRTPKEY  419

MtyB  419  REETYLTSPVFNRGTCNEEGRAATPPIQERAYKVYKERIESFELPDMTQAQKDLLNEHLP  478
           RE+ YL S VFNR  C E  R     I++RA KVY  R+E++ELPD T  QK LLN  LP
MtcB  420  REDNYLASEVFNRKGCKENTREEQGDIRDRARKVYDARMEAYELPDTTLEQKKLLNTELP  479

MtyB  479  EKFKIK  484
           E++K
MtcB  480  EQYKFDI 486
```

Figure S6. BLAST sequence alignment of MtyB, the predominant methyltransferase found in γ-butyrobetaine-grown *E. limosum* and MtcB, the predominant methyltransferase found in *L*-carnitine-grown cells.

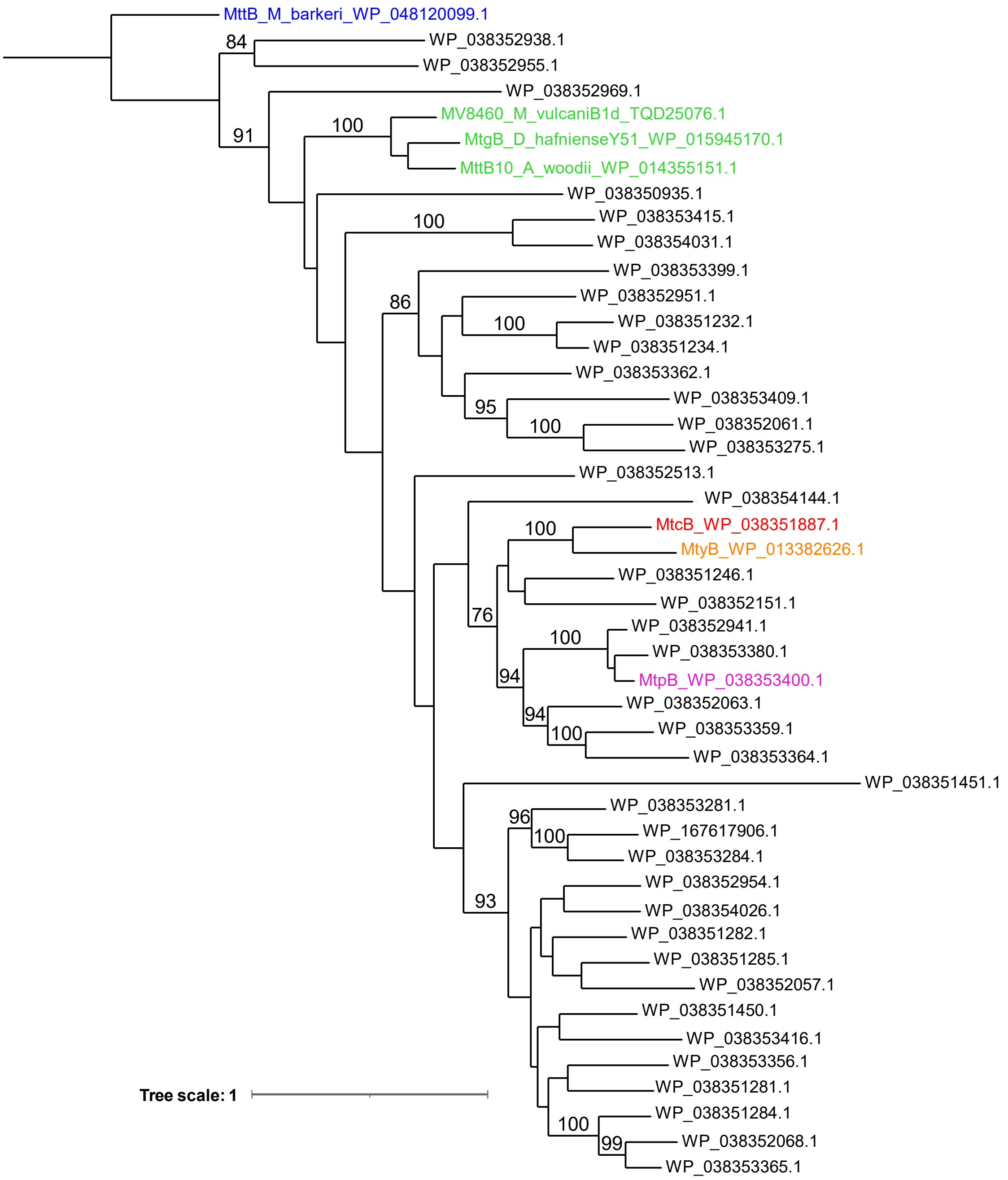

Figure S7. Maximum likelihood phylogeny of non-pyrrolysine MttB homologs. Maximum likelihood phylogeny (1000 bootstrap replicates, bootstrap values ≥80 as indicated) constructed using MEGA (59) of forty-two predicted non-pyrrolysine MttB homologs encoded in the genome of *Eubacterium limosum* ATCC 8486, including the characterized MtyB (orange), MtcB (red), and MtpB (pink). Also included is MtgB (the glycine betaine dependent enzyme from *Desulfitobacterium hafniense*) and its homologs MttB10 from *Acetobacterium woodii* and MV8460 from *Methanolobus vulcani* B1d (all in green). The pyrrolysine-containing MttB (blue) from *Methanosarcina barkeri* was used to root the tree.
